# Humoral response to neurofilaments and dipeptide repeats in ALS progression

**DOI:** 10.1002/acn3.51428

**Published:** 2021-07-27

**Authors:** Fabiola Puentes, Vittoria Lombardi, Ching‐Hua Lu, Ozlem Yildiz, Pietro Fratta, Adrian Isaacs, Yoana Bobeva, Joanne Wuu, ALS Biomarker Consortium, CReATe Consortium, Michael Benatar, Andrea Malaspina

**Affiliations:** ^1^ Neurodegeneration Group Blizard Institute, Barts and the London School of Medicine and Dentistry Queen Mary University of London London E1 2AT United Kingdom; ^2^ Department of Neuromuscular Diseases UCL Queen Square Institute of Neurology University College London London WC1N 3BG United Kingdom; ^3^ School of Medicine China Medical University 91 Xueshi Road North District Taichung City 404 Taiwan; ^4^ Department of Neurology University of Miami Miami Florida USA

## Abstract

**Objective:**

To appraise the utility as biomarkers of blood antibodies and immune complexes to neurofilaments and dipeptide repeat proteins, the products of translation of the most common genetic mutation in amyotrophic lateral sclerosis (ALS).

**Methods:**

Antibodies and immune complexes against neurofilament light, medium, heavy chains as well as poly‐(GP)‐(GR) dipeptide repeats were measured in blood samples from the ALS Biomarkers (*n* = 107) and the phenotype–genotype biomarker (*n* = 129) studies and in 140 healthy controls. Target analyte levels were studied longitudinally in 37 ALS cases. Participants were stratified according to the rate of disease progression estimated before and after baseline and *C9orf72* genetic status. Survival and longitudinal analyses were undertaken with reference to matched neurofilament protein expression.

**Results:**

Compared to healthy controls, total neurofilament proteins and antibodies, neurofilament light immune complexes (*p* < 0.0001), and neurofilament heavy antibodies (*p* = 0.0061) were significantly elevated in ALS, patients with faster progressing disease (*p* < 0.0001) and in ALS cases with a *C9orf72* mutation (*p* < 0.0003). Blood neurofilament light protein discriminated better ALS from healthy controls (AUC: 0.92; *p* < 0.0001) and faster from slower progressing ALS (AUC: 0.86; *p* < 0.0001) compared to heavy‐chain antibodies and light‐chain immune complexes (AUC: 0.79; *p* < 0.0001 and AUC: 0.74; *p* < 0.0001). Lower neurofilament heavy antibodies were associated with longer survival (Log‐rank Chi‐square: 7.39; *p* = 0.0065). Increasing levels of antibodies and immune complexes between time points were observed in faster progressing ALS.

**Conclusions:**

We report a distinctive humoral response characterized by raising antibodies against neurofilaments and dipeptide repeats in faster progressing and *C9orf72* genetic mutation carriers ALS patients. We confirm the significance of plasma neurofilament proteins in the clinical stratification of ALS.

## Introduction

The lack of individualized biomarkers that can be used to define the future course of the disease and measure treatment response is holding back therapeutic development in amyotrophic lateral sclerosis (ALS), a fatal neurodegenerative disorder.^1^ Axonal proteins like neurofilaments (Nf) have recently emerged as biomarkers with potential prognostic and pharmacodynamic utility in ALS.^1^, ^2^, ^3^, ^4^, ^5^, ^6^, ^7^, ^8^, ^9^ After an initial increase in the prodromal and early stages of ALS, Nf concentrations remain largely stable, against the observed remarkable variability in the rate of disease progression and the expected cumulative burden of axonal loss.^7^, ^9^, ^10^ Understanding the biological processes that regulate Nf homeostasis in blood, including the endogenous antibody response which clears these proteins from the circulation, is a critical step toward implementing the use of these biomarkers in clinical trials for ALS.

Disease progression is increasingly seen as dependent on ALS‐associated genetic alterations like the intronic expansion of the *C9orf72* gene. Clinical heterogeneity in ALS has also been linked to concerted innate and adaptive immunological responses, including the reduction of circulating T regulatory cells (Tregs) in ALS individuals with reduced survival.^11^ Loss of Tregs function is thought to lead to diminished self‐immune tolerance and to raise the levels of endogenous antibodies (Abs) and immune complexes (ICs) to self‐proteins, including Nf.^12^, ^13^, ^14^ A state of enhanced autoimmunity to self‐proteins like the Nf isoforms light (Nf‐L), medium (Nf‐M), and heavy (Nf‐H) but also to dipeptide repeats (DPR), the product of translation of the mutated *C9orf72* gene, may therefore result from a changing self‐tolerance environment in ALS. We have already shown Nf‐L Abs reaching a peak in blood at a late stage of ALS, and how the concentration of these Abs correlates with functional impairment and survival.^10^ Nf‐ and DPR‐specific humoral responses could, therefore, be investigated as potential disease progression biomarkers and tested for the clinical stratification of ALS. Their concentration in blood may also be informative as pharmacodynamic biomarker in clinical trials where faster progressing ALS and individuals with the *C9orf72* genetic mutation (C9+ve ALS) have been recently singled out for their response to high‐caloric diet and gene‐modifying treatments.^15^

The advantage of blood Nf and DPR Abs used as biomarkers is also their accessibility. Neurofilament light (Nf‐L) and DPR protein concentrations are much higher in CSF compared to blood, reflecting a brain, CSF and blood concentration gradient and the significantly reduced Nf‐ and DPR‐specific intrathecal humoral responses.^16^, ^17^ In contrast, blood is more enriched with Abs and ICs and accessible through minimally invasive procedures.

Here, we appraise the relevance of the humoral response to Nf and DPR as potential biomarker for the clinical stratification of ALS.

## Patients and Methods

### Subjects

ALS patients fulfilling standard diagnostic criteria for ALS diagnosis^18^ from the ALS biomarkers study cohort (ABC, London, UK) and from the Clinical Research in ALS and Related Disorders for Therapeutic Development (CReATe) Consortium’s Phenotype Genotype Biomarker (PGB) study (NCT02327845) were consented in the study. Two separate cohorts of age‐ and sex‐matched healthy controls from the United Kingdom were also consented in the study (ethical approval for UK, REC‐reference 09/H0703/27 and for PGB, University of Miami central IRB––CReATe Consortium). Exclusion criteria for both ALS and healthy controls (HC) included neuroinflammatory or neurodegenerative disorders, recent injuries, systemic or organ‐specific autoimmune disorders, recent treatment with steroids, immunosuppressants, or immunoglobulins.

All participants were sampled at baseline, while a subset of individuals from ABC was sampled serially every 3–6 months for longitudinal analysis. The ALS functional rating scale‐revised (ALSFRS‐R) score was recorded at each visit. Genetic analysis for the *C9orf72* mutation was undertaken for all ALS cases. C9+ve ALS cases were included in both ABC and PGB.

### Plasma extraction

Blood samples were drawn by venepuncture, collected in anticoagulant (EDTA)‐coated tubes (BD vacutainer, UK) and centrifuged at 1300 *g* for 10 min for the ABC patients and at 1750 *g* for the PGB samples at room temperature (RT). Plasma was aliquoted in polypropylene tubes (Nunc, UK) and stored at −80℃ (ABC) and at −70℃ (PGB) until use.

### Proteins and peptides

For Abs detection, the following capture proteins were selected and coated onto ELISA plates: human‐recombinant Nf‐L (MyBioSource, USA), bovine Nf‐L, Nf‐M, Nf‐H (Progen, Germany), and 20 amino acid‐long biotinylated peptides (Poly‐(GA)10, poly‐(GP)10, and poly‐(GR)10) for DPR,^19^, ^20^ synthesized at the National Physical Laboratory UK (biotinylated at the amino terminal region with purity >90%).

Selection of bovine neurofilaments proteins (Nf‐L 68 KDa, Nf‐M 160 KDa, and Nf‐H 200 KDa; Progen) was based on availability (e.g., lack of suitable human recombinant proteins for Nf‐M and Nf‐H), high homology to human proteins (80%–98.3% identity for Nf‐M and Nf‐H, respectively), detection of a single band at the expected molecular weight on electrophoresis, and purity >98%. To account for the expected Abs cross‐reactivity to different Nf chains, we tested both individual response and (total) reactivity to all Nf isoforms combined.

### Quantitative antibody determination

Plasma samples were tested for antibody detection by enzyme‐linked immunosorbent assay (ELISA) as previously reported.^21^ Nunc‐Immuno microtiter 96‐well solid plates (Thermo Fisher Scientific, UK) were coated with 2 μg/mL of recombinant human Nf‐L (MyBioSource, USA) or 3 μg/mL of bovine Nf‐L, Nf‐M, or Nf‐H (Progen, Germany) in carbonate buffer pH 9.6, overnight at 4℃. Plates were blocked with 2% bovine serum albumin PBS/BSA and plasma was added at 1:100 dilution in triplicates. After 1‐h incubation, antibody binding was detected with horseradish peroxidase (HRP)‐conjugated goat anti‐human IgG (Sigma, UK; 1:5000 dilution). The reaction was developed with TMB (3,3′,5,5′‐tetramethylbenzidine substrate, Thermo Fisher Scientific, UK) and stopped by the addition of 2 mol/L hydrochloric acid (Fluka, UK).

Neutravidin plates (Thermo Fisher Scientific, UK) were incubated with 10 μg/mL of biotinylated poly‐(GP), poly‐(GR), and poly‐(GA) DPR peptides in Tris‐buffered saline (25 mmol/L Tris, 150 mmol/L NaCl pH 7.2, 0.1% BSA, and 0.05% Tween‐20) followed by incubation with plasma and washing with TBS. Bound IgG was detected by the addition of enzyme‐labeled secondary Abs as described above. The reaction was measured in a Synergy HT microplate reader (BioTek instruments, VT).

### Quantitative immune complexes determination

Quantification of neurofilament and DPR ICs was carried out by ELISA as previously reported.^22^ Microtiter 96‐well plates (Thermo Fisher Scientific, UK) were coated overnight at 4℃ with 0.1 mL of 0.5 µg/mL rabbit polyclonal anti‐human Nf‐L (Abbexa, UK), anti‐Nf‐M (Abbexa, UK), anti‐Nf‐H (Novus Biological, UK), or polyclonal rabbit anti‐(GP) and anti‐(GR) Abs (kindly provided by Professor Adrian Isaac, UCL, UK). All Abs were diluted in carbonate buffer pH 9.6. After blocking with PBS and 2% BSA, plates were incubated with plasma samples (1:100 dilution) in triplicate for 1 h at RT. After washing with PBS 0.1% Tween‐20, (HRP)‐conjugated goat anti‐human IgG (Sigma, UK) was added and incubated for 1 h at RT. The reaction was developed with TMB and measured at 450 nm.

### Inclusion of standard curves to improve the accuracy of immunoassays

Plates were pre‐coated with anti‐human IgG (Fc‐specific) antibody (Sigma, UK) to capture purified human IgG (Sigma, UK) which was serially diluted (1:2) from 375 to 0.02 ng/mL to generate a standard calibration curve. After incubation with peroxidase‐conjugated goat anti‐human IgG (whole molecule), TMB substrate was added for detection. Optical densities were normalized by subtracting the absorbance derived from uncoated wells. Plasma Abs and the proportional amount of IgG and ICs (pg/mL) were quantified by interpolation of optical densities from the standard curve. Plasma samples from ALS patients and HC were equally distributed on each plate and measured in duplicate.

### Measurement of plasma Nf‐L and Nf‐H levels

Analysis of Nf‐L and Nf‐H protein expression in plasma was undertaken by single‐molecule array (Simoa) using a digital immunoassay HD‐1 Analyzer (Quanterix, Lexington, MA). Standards, primary and secondary Abs, detection range including lower and upper limits of detection were specified in the manufacturer’s conditions of the commercial assays. Analysis of Nf‐M protein isoform was not attempted as currently available immunoassays have not been extensively tested in ALS.^23^

Genotyping of the *C9orf72* gene in the ALS population in this study was performed as previously reported.^24^

### Statistical analysis

Symptom onset was defined as the first occurrence of muscle weakness, involving the limbs, bulbar musculature (dysarthria or dysphagia), or respiratory muscles. Survival was defined as time from symptom onset to permanent assisted ventilation (≥22 h/d noninvasive ventilation), tracheostomy, or death. We calculated the estimated rate of disease progression at baseline by subtracting the ALSFRS‐R from 48 and dividing by the time from reported first symptoms of disease to baseline (ΔFRS, points/month). The post‐baseline rate was obtained subtracting the last recorded ALSFRS‐R score from the baseline ALSFRS‐R divided by the time interval (ALSFRS‐R change). A faster progressing ALS (ALS‐F) was defined as ΔFRS and ALSFRS‐R change greater than −1.0 points/month while slower progressing ALS (ALS‐S) as ΔFRS and ALSFRS‐R change less than −0.5 points/month.

Continuous variables were expressed as median (interquartile range; IQR) or mean (standard deviation, SD); nonparametric group analysis was performed using Kruskal–Wallis one‐way test of variance on ranks and after log transformation of raw values. Pairwise correlation analysis (Pearson’s) was also used to test the associations between analyte concentrations and functional scores.

To investigate longitudinal trajectories of the plasma analytes in faster, slower progressing ALS and in C9+ve ALS patients, we used ANOVA for repeated measures fitting a mixed model with missing values at random. For each marker, we obtained a box plot representation of concentration levels at each consecutive time point. The changes in the markers longitudinal expression were also visualized using spaghetti plots. We then calculated the change in concentration between last and early time point of Abs and ICs divided for the time interval. Nf, DPR, and their Abs and ICs concentration values were not normally distributed hence natural logarithm transformation was used in the analyses.

Receiver operating characteristic (ROC) curve nonparametric analyses and their corresponding 95% confidence intervals (CIs) were calculated to evaluate the discrimination among phenotypic variants and between ALS and HC, based on Abs and ICs levels.

Univariate and multivariate survival analyses were undertaken using Kaplan–Meier curves––log‐rank test and Cox proportional hazards models. Multivariate analysis was performed incorporating clinical variables that have been previously associated with survival in ALS including sex, age, ALSFRS‐R, and bulbar onset.

The intra‐assay coefficients of variation (CVs) were calculated by dividing the standard deviations (SD) of a set of measurements by the set mean and multiplying by 100. The inter‐assay CVs to assess plate‐to‐plate consistency were calculated as the variance between runs of standard sample replicates on different plates. The inter‐assay CV (% CV) was expressed as SD of plate means ÷ mean of plate means × 100.

A *p* value of less than 0.05 was considered to be statistically significant. Data were analyzed using Prism (Version 8.0, GraphPad Software, San Diego, CA, USA) and SPSS.

### Data availability

Anonymized data about the ALS biomarkers study can be requested from the corresponding author. Requests for access to data from the CReATe Consortium should be directed to ProjectCReATe@miami.edu.

## Results

### Study population

Demographic and clinical characteristics for the cross‐sectional and longitudinal cohorts are summarized in (Tables 1 and 2), respectively. The ABC included 107 ALS patients (45 ALS‐F, 36 ALS‐S, and 26 C9+ve ALS; mean age at baseline: 61.9 (SD ± 10.7)) while the PGB cohort included 129 ALS patients (55 ALS‐F, 54 ALS‐S, and 20 C9+ve ALS; mean age at baseline: 58.6 (SD ± 11.57)). A group of 76 healthy controls (HC‐1; mean age at sampling: 58.6 ± 8.5) and a second group of 64 HC (HC‐2; mean age at sampling: 60.5 ± 0.2) were selected to match the ABC and PGB cohorts. The median latency (months) and interquartile range (IQR) from disease onset to diagnosis for the ABC cohort were 38.5 (5.6–92.8) for ALS‐S, 12.3 (2.3–27) for ALS‐F, and 13.4 (12.6–26.2) for C9+ve ALS patients, while for the PGB cohort, 10.9 (1.05–30.8) for ALS‐S, 6.1 (0.3–25.3) for ALS‐F, and 9.8 (1.1–24.9) for the C9+ve ALS subset of patients (Table 1).

**Table 1:** Cross‐sectional study. Demographic and clinical features of the ABC and PGB cohorts.

	ALS‐slower		ALS‐faster		C9+ve ALS		Healthy controls	
	ABC	PGB	ABC	PGB	ABC	PGB	HC‐1	HC‐2
	*N* = 36	*N* = 54	*N* = 45	*N* = 55	*N* = 26	*N* = 20	*N* = 76	*N* = 64
Male (%)	23 (64%)	29 (54%)	22 (40%)	28 (51%)	13 (50%)	9 (45%)	27 (36%)	26 (41%)
Age at baseline, years								
Mean (±SD)	63.4 ± 9.4	57.1 ± 13.1	63.3 ± 11.3	61.0 ± 10.7	57.2 ± 10.4	56.35 ± 8.8	58.7 ± 8.5	61.5 ± 9.3
Site of disease onset								
Bulbar	7	9	18	19	9	7	(n/a)	
Limb	29	46	22	36	15	13	(n/a)	
Bulbar and Limb	0	0	0	0	0	0	(n/a)	
Other**	1	5	3	7	2	2	(n/a)	
Time from onset to diagnosis, months								
Median (interquartile range)	38.5 (5.6–92.8)	11.0 (1.05–30.86)	12.3 (2.34–27)	6.2 (0.33–25.31)	13.4 (12.6–26.28)	9.9 (1.12–24.9)	(n/a)	
Baseline ALSFRS‐R								
Mean (±SD)	38.7 ± 7.6	–	32.9 ± 7.6	–	35.4 ± 8.9	–	(n/a)	
Survival, months								
Median (interquartile range)	63.8 (28.4–114.3)	41.6 (15.0–85.0)	23.3 (3.4–53.5)	34.0 (19.2–38.0)	28.8 (7.8–73.3)	29.4 (8.5–21.8)	(n/a)	
Baseline ΔFRS (points/month)								
Mean (±SD)	−0.25 ± 0.14	−0.43 ± 0.21	−1.11 ± 0.79	−1.17 ± 0.97	−0.81 ± 0.72	−0.90 ± 0.68	(n/a)	
ALSFRS‐R slope (points/months)								
Mean (±SD)	−0.2 ± 0.2	−0.2 ± 0.2	−1.5 ± 1.2	−1.7 ± 0.8	−1.2 ± 0.9	−1.1 ± 0.9	(n/a)	

ALSFRS‐R, Amyotrophic lateral sclerosis functional rating scale‐revised; ABC, ALS Biomarkers Study Cohort; PGB, phenotype–genotype biomarker (PGB) study; Other**, onset in other regions (e.g., respiratory); C9+ve ALS, ALS patients with a mutation in the *C9orf72* gene; Baseline ΔFRS (points/month), estimated ALSFRS‐R rate of progression from onset (assumed score 48) to baseline visit; ALSFRS‐R change (in points/month), ALSFRS‐R rate of progression calculated using ALSFRS‐R scores collected at longitudinal study visits (post‐baseline slope); Survival, survival duration from symptom onset to permanent assisted ventilation/tracheostomy, death, or censoring. HC‐1, HC‐2, healthy control groups

**Table 2:** Demographic and clinical features of ALS cohorts used in the longitudinal study.

Longitudinal data	ALS‐slower	ALS‐faster	C9orf72 ALS
Number	15	11	11
Male (%)	11 (73%)	7 (64%)	6 (55%)
Age at baseline, years			
Mean (±SD)	64.3 ± 8.9	66.2 ± 7.7	58.3 ± 11.9
Site of disease onset			
Bulbar	3	5	5
Limb	12	5	6
Bulbar and Limb	0	0	0
Other**	0	1	0
Time from onset to diagnosis, months			
Median (interquartile range)	3.40 (2.27–5.80)	1.10 (0.88–1.52)	1.53 (1.24–2.34)
Baseline ALSFRS‐R			
Mean (±SD)	42.73 ± 4.20	36.91 ± 7.16	38.64 ± 5.41
Survival, months			
Median (interquartile range)	89.8 (84.8–107.2)	29.8 (21.0–31.5)	35.2 (29.1–48.5)
Baseline ΔFRS (points/month)			
Mean (±SD)	−0.20 ± 0.12	−1.01 ± 0.48	−0.93 ± 0.72
ALSFRS‐R slope (points/month)			
Mean (±SD)	−0.24 ± 0.18	−1.83 ± 0.96	−1.08 ± 0.89
Duration of longitudinal follow‐up, months			
Median (interquartile range)	49.7 (41.41–63.3)	5.93 (3.4–9.58)	10.97 (5.65–16.85)
N (%) with four time points	13 (86.6%)	2 (20%)	9 (64.28%)
N (%) with three time points	2 (13.3%)	7 (70%)	3 (21.4%)

ALSFRS‐R, Amyotrophic lateral sclerosis functional rating scale‐revised; Other**, onset in other regions (e.g., respiratory); Baseline ΔFRS (points/month), estimated/reported ALSFRS‐R rate of progression from onset (assumed score 48) to baseline visit (ALSFRS‐R slope/months from symptoms onset to baseline); ALSFRS‐R change (in points/month), ALSFRS‐R rate of progression calculated using ALSFRS‐R scores collected at longitudinal study visits (Post‐baseline slope); Survival, survival duration from onset to permanent assisted ventilation/tracheostomy‐free, or censoring (in months).

Fifteen ALS‐S, 11 ALS‐F, and 11 C9+ve ALS patients from the ABC were studied longitudinally in up to 4 distinct time points (Table 2). Follow‐up visits were on average at 3–8 months intervals. Disease onset was in the limbs in 61.7 and 73.6% of ALS patients in the ABC and PGB cross‐sectional cohorts, and in 62.1% of the longitudinal cohort; the rest had predominantly bulbar onset disease. The average baseline ALSFRS‐R score was 37.4 ± 6.8 for the ABC, 37.5 ± 5.8 for the PGB cohort, and 40.3 ± 6.2 for the longitudinal cohort (Tables 1 and 2).

### Antibody and immune complex assay performance

The intra‐assay CVs were 9.3%, 9.9%, 11.2%, 6.8%, and 5.0% for the Nf‐L, Nf‐M, Nf‐H, GP, and GR antibody assays, respectively. The inter‐assay CVs were 18.1%, 9.9%, 14.6%, 10.4%, and 13.4% for the detection of Nf‐L, Nf‐M, Nf‐H, GP, and GR Abs, respectively. For immune complex assays, the intra‐assay CVs were 11.2%, 10.6%, 8.7%, 11.1%, and 10.0% for Nf‐L, Nf‐M, Nf‐H, GP, and GR, respectively. The inter‐assay CVs were 16.0%, 14.3%, 12.7%, 10.3%, and 10.8% for Nf‐L, Nf‐M, Nf‐H, GP, and GR ICs, respectively. The lower limit of detection (LLD) across experiments was 0.058 ng/mL and the upper limit of detection was 280 ng/mL for Abs assays, and 0.03 ng/mL and 65 ng/mL, respectively, for ICs. Missing data (i.e., technical replicates below LLD) were 2.3% for Abs and 3.2% for ICs of all assays.

### Humoral response to total neurofilaments (Nf) and dipeptide repeats (poly‐DPR)

**Figure 1:**
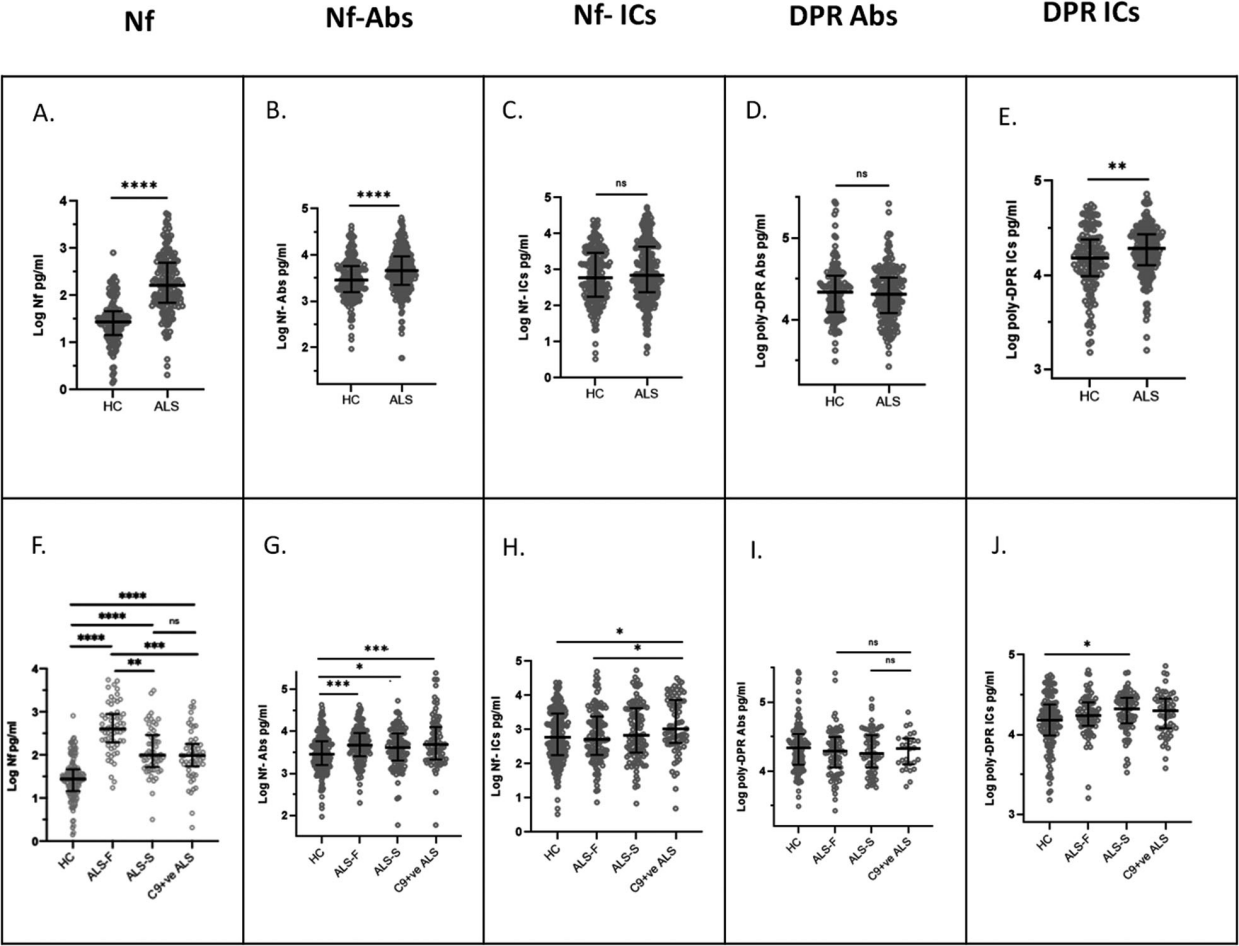
Neurofilament (Nf) levels and immune response to Nf and dipeptide repeat (DPR) peptides in blood samples from the ALS Biomarkers Cohort (ABC) and from healthy controls. (A) Nf (neurofilament light (Nf‐L) and heavy chain (Nf‐H) isoforms). (B) Antibodies against Nf‐L, Nf‐M, and Nf‐H (Nf‐Abs). (C) Nf‐L, Nf‐M, and Nf‐H immuno complexes (Nf‐ICs). (D) poly‐(GP) and poly‐(GR) variants of dipeptide repeats (poly‐DPR Abs) Abs and (E) poly‐(GP) and poly‐(GR) variants of immuno complexes (poly‐DPR ICs). Nf and Nf‐Abs levels are significantly higher in ALS compared to HC (A and B, *p* < 0.0001). No differences in Nf‐ICs and poly‐DPR Abs levels are observed between ALS and HC (C and D), while plasma poly‐DPR ICs are higher in ALS patients compared to HC (E, *p* < 0.01). Nf are higher in ALS‐F compared to HC (*p* < 0.0001), ALS‐S (*p* = 0.002), and C9+ve ALS (*p* < 0.001). Nf are also elevated in ALS‐S and C9+ve ALS compared to HC (F, *p* < 0.0001). Nf Abs plasma concentration in C9+ve ALS and ALS‐F is elevated compared to HC and ALS‐S (G*, p* < 0.001). Higher concentration of Nf‐ICs in C9+ve ALS compared to HC (*p* < 0.05) and ALS‐F (H*, p* < 0.05). poly‐DPR ICs plasma levels are increased in ALS‐S compared to HC (I*, p* ≤ 0.05).

Plasma concentrations of neurofilament proteins (Nf‐L and Nf‐H) and Nf Abs (Nf‐L, Nf‐M, and Nf‐H Abs) were elevated among ALS patients compared to HC‐1 (Fig. 1A and B; *p* < 0.0001). There was, however, no difference in the level of Nf (Nf‐L, Nf‐M, and Nf‐H ICs) ICs in ALS patients compared to HC‐1 (Fig. 1C). In contrast, ICs, but not Abs against DPRs, were increased in ALS compared to HC (Fig. 1D and E, *p* = 0.0029).

Blood Nf proteins (Nf‐L and Nf‐H) were also elevated in all ALS subgroups (ALS‐F, ALS‐S, and C9+ve ALS) compared to HC‐1 (Fig. 1F, *p* < 0.0001), with the highest level of expression in ALS‐F compared to HC (*p* < 0.0001), ALS‐S (*p* = 0.002), and C9+ve ALS (Fig. 1G, *p* = 0.0001). Nf levels in C9+ve ALS were comparable to those in the slower progressing ALS group, which may reflect the intermediate rate of progression in this subset of patients compared with faster or slower progressing ALS cases (Table 1).

Nf‐Abs were similarly elevated in the ALS subgroups compared to HC1. ALS‐F and C9+ve ALS showed the highest Nf‐Abs plasma concentrations compared to HC‐1 (Fig. 1G, *p* = 0.0009 and *p* = 0.0001, respectively).

Nf‐ICs levels were higher in C9+ve ALS patients compared to HC‐1 and ALS‐F (Fig. 1H, *p* = 0.022 and *p* = 0.030), suggesting that the expression of this marker may be influenced by the C9orf72 genetic status and not by the rate of disease progression. No difference in poly‐DPR Abs expression between ALS subgroups and HC‐1 was observed (total poly‐DPR Abs, Fig. 1I), while poly‐DPR ICs were higher in ALS‐S compared to HC (Fig. 1J, *p* = 0.02).

### Humoral response to neurofilament isoforms

Group analysis and ROC were used to examine cross‐sectionally how Abs and ICs to Nf and poly‐DPR discriminated ALS and its phenotypic variants from HC, compared to Nf‐L and Nf‐H proteins measured in the same plasma samples. We have previously reported the absence of differential regulation of plasma Nf‐M between ALS and HC using a commercially available assay.^23^

**Figure 2:**
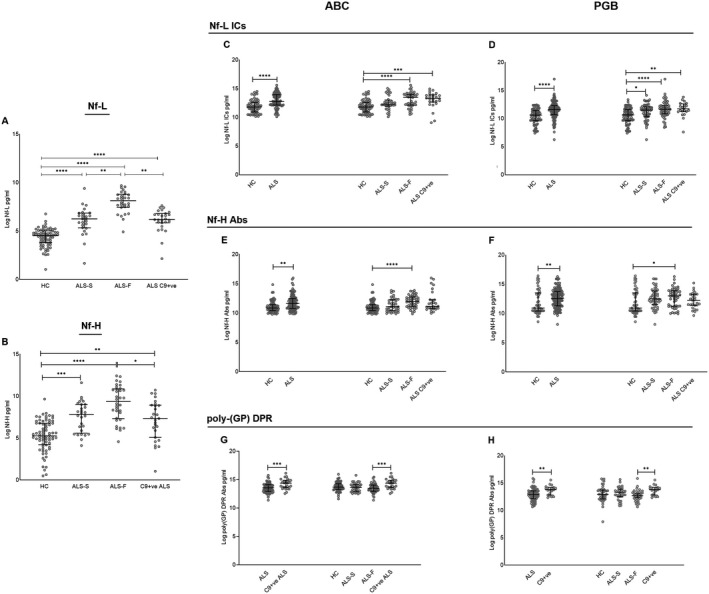
Nf‐L and Nf‐H protein levels and humoral response to Nf‐L, Nf‐H, and poly‐GP dipeptide repeats (DPR) in phenotypic variants of ALS, in ALS individuals with a C9orf72 mutation (C9+ve) and in healthy controls. Group analysis comparing Nf‐L and Nf‐H protein isoforms concentration, Nf‐L, Nf‐M, and Nf‐H antibodies (Abs) and immuno complexes (ICs) concentration in phenotypic (ALS‐F and ALS‐S) and genotypic (C9+ve) variants of ALS from the ALS biomarkers study (ABC), phenotype–genotype biomarker study (PGB) cohort, and from two groups of healthy controls (HC‐1 and HC‐2, Table 1). Kruskal–Wallis one‐way analysis was used for multiple comparisons. The scatter dot plots show the median with interquartile range. The statistical difference between groups is shown as (****p < 0.0001), (***p < 0.001), (**p < 0.01), (*p ≤ 0.05) or as non‐significant (n.s). (A) Nf‐L protein levels: ABC cohort and HC‐1. (B) Nf‐H protein levels: ABC cohort and HC‐1. (C and D) Nf‐L ICs levels: ABC and PGB cohorts compared to HC‐1 and HC‐2, respectively. (E and F) Nf‐H Abs levels: ABC and PGB cohorts compared to HC‐1 and HC‐2, respectively. (G and H) Poly‐(GP) DPR Abs levels: ABC and PGB cohorts compared to HC‐1 and HC‐2, respectively.

Consistent with previous reports, plasma concentrations of Nf‐L and Nf‐H proteins were increased among ALS patients compared to HC‐1 (*p* < 0.0001; Fig. 2A and B) and significantly elevated in ALS‐F and ALS‐S compared to HC‐1 (*p* < 0.0001 and *p* = 0.0014; Fig. 2A).^8^, ^25^ Nf‐L plasma concentration was greater in ALS‐F compared to ALS‐S (*p* = 0.001; Fig. 2A).^8^ Blood concentrations of Nf‐L and Nf‐H proteins in C9+ve ALS patients were comparable to those detected in ALS‐S and reduced compared to the ALS‐F subgroup (Fig. 2A and B; *p* = 0.002 and *p* = 0.027, respectively).

Plasma Nf‐L ICs were significantly elevated in both ABC and PGB compared to HC‐1 and HC‐2, respectively (*p* < 0.0001; Fig. 2C and D), while among ALS variants, the highest levels of Nf‐L ICs blood concentration were found in ALS‐F and in C9+ve ALS patients compared to HC, in both ABC (*p* < 0.0001 and *p* = 0.0003) and PGB (*p* < 0.0001 and *p* = 0.0025), and in ALS‐S compared to HC (PGB cohort, *p* = 0.015; Fig. 2C and D).

Plasma Nf‐H Abs were elevated in ALS patients in both ABC (*p* = 0.0023) and PGB (*p* = 0.01) compared to HC and had higher expression levels in ALS‐F compared to HC in both ABC (*p* < 0.0001) and PGB (*p* < 0.05) (Fig. 2E and F).

Nf‐M Abs were upregulated in ALS (*p* = 0.033) compared to HC‐1 and in ALS‐F patients compared to HC‐1 (*p* = 0.047; Fig. S1).

**Figure 3:**
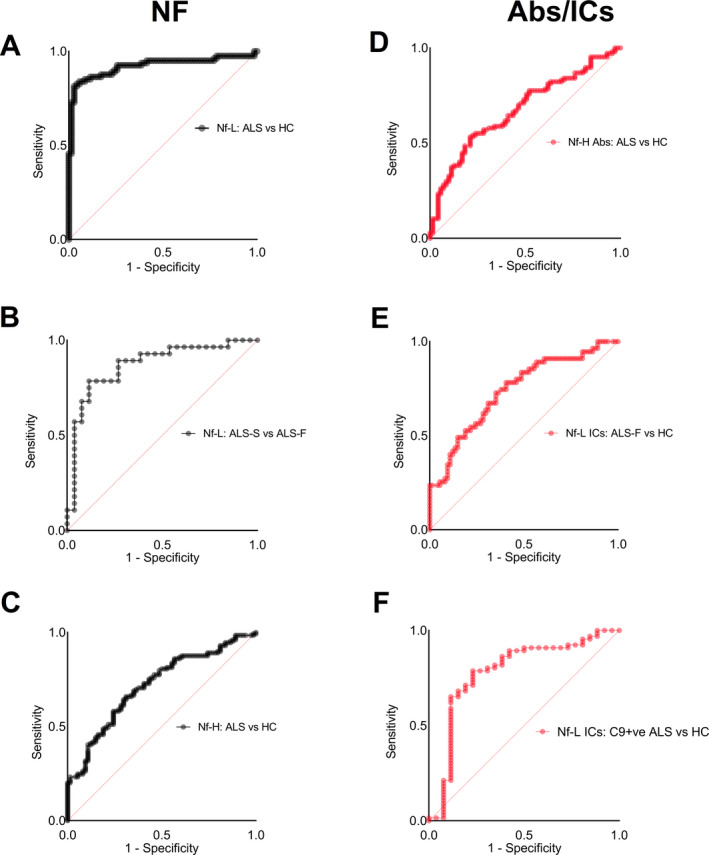
Receiver operating characteristic (ROC) nonparametric analysis to evaluate the performance of Nf and Abs/ICs analytes in the discrimination of ALS from healthy controls and phenotypic variants of the disease (plots indicate sensitivity against 1‐specificity). (A) Nf‐L protein plasma levels strongly discriminate ALS patients from HC (AUC = 0.93, *p* < 0.0001) and (B) ALS‐F from ALS‐S (AUC = 0.89, *p* < 0.0001). (C) Nf‐H proteins plasma levels provide a moderate separation of ALS from HC (AUC = 0.72, respectively, *p* = 0.0001). (D) Nf‐H Abs expression levels separate ALS from HC (AUC: 0.68; *p* < 0.0001). (E) Nf‐L ICs expression shows a fair discrimination of ALS‐F from C9+ve ALS (AUC = 0.78, *p* < 0.0001) and (F) ALS‐F from HC (AUC = 0.72; *p* < 0.0001).

Analysis of sensitivity and specificity using ROC indicated that Nf‐L and Nf‐H blood proteins performed well in the separation of ALS from HC (Fig. 3A, area under the curve (AUC) = 0.93 and Fig. 3C, AUC = 0.87, *p* < 0.0001), while Nf‐L (but not Nf‐H) discriminated ALS‐F from ALS‐S (Fig. 3B, AUC = 0.86, *p* < 0.0001).

Among Abs and ICs, Nf‐H Abs revealed the best performance in distinguishing ALS from HC (ABC; Fig. 3D, AUC = 0.72, respectively, *p* = 0.0001), while plasma Nf‐L ICs discriminated C9+ve from HC (Fig. 3F, AUC = 0.78, *p* < 0.0001) and ALS‐F from HC (Fig. 3E, AUC = 0.72; *p* < 0.0001).

Nf‐M Abs concentrations were able to discriminate ALS‐F from HC (AUC = 0.64, *p* = 0.011) and ALS from HC (AUC = 0.63, *p* = 0.0056; Fig. S1B).

### Plasma poly‐(GP) DPRs Abs and IC in ALS variants

Plasma levels of Abs and ICs to DPR were investigated in ALS subgroups, including C9+ve ALS patients and those without the *C9orf72* mutation (C9‐ve). The concentration of poly‐(GP) DPR Abs was elevated in blood from C9+ve ALS cases compared to C9‐ve ALS patients in both ABC (*p* = 0.0008) and PGB (*p* = 0.0033). Poly‐(GP) DPR Abs showed a modest discrimination of C9+ve from C9‐ve ALS (AUC = 0.72, *p* = 0.0008). Levels of poly‐(GA) DPR Abs were not different among the ALS subgroups, neither discriminated ALS from HC (data not shown).

### Survival analysis

**Figure 4:**
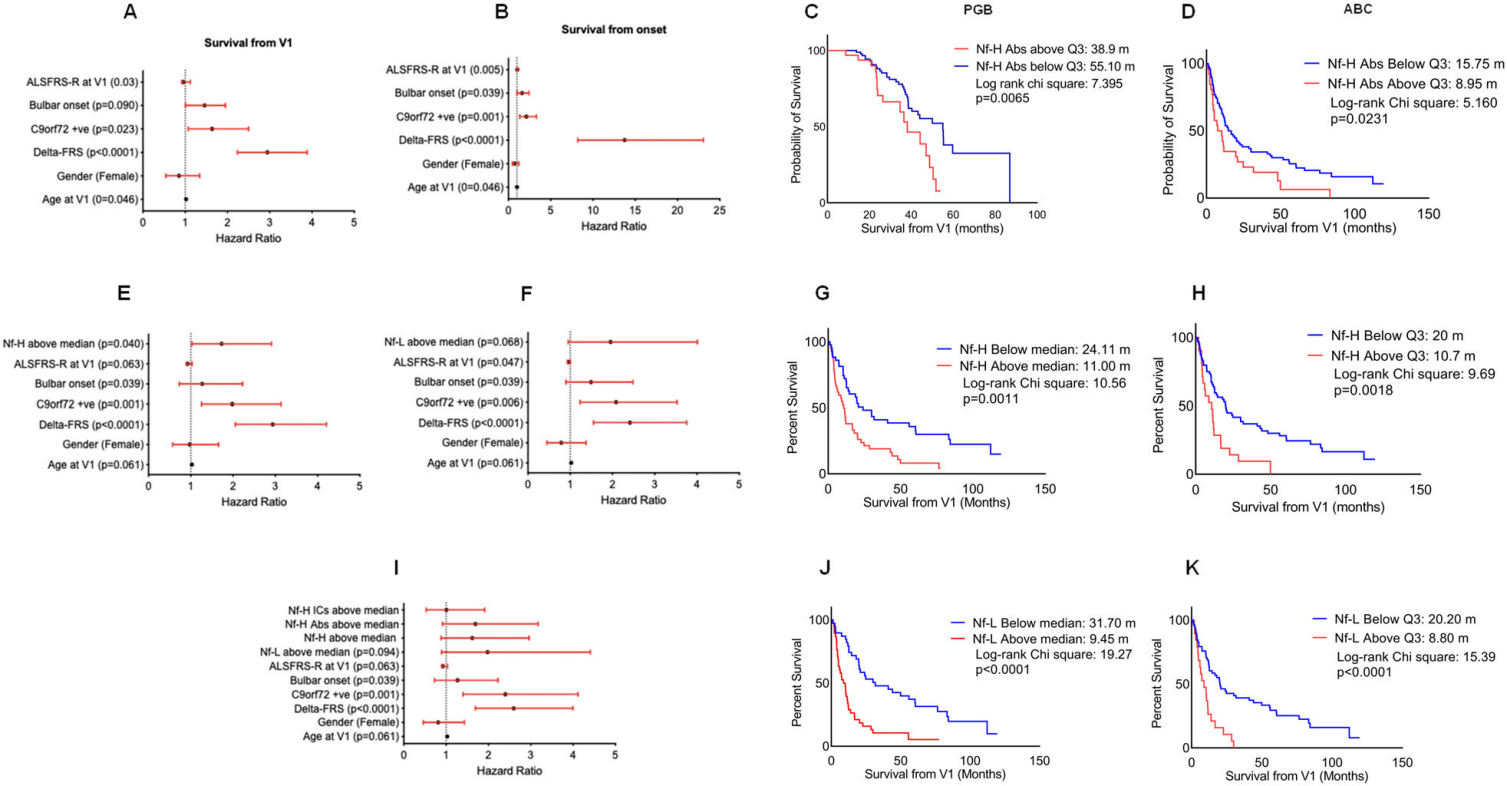
Analysis of Nf‐H, Nf‐L, and Nf‐H Abs plasma levels and survival: mortality hazard ratio and Kaplan–Meier survival analysis. Multivariate Cox regression analysis was calculated from baseline (Visit 1: V1) and from onset (A and B) incorporating in the model age at V1, ΔFRS, ALFRS‐R at V1, C9+ve status, and bulbar onset of disease as clinical variables. Higher plasma concentrations (above median) of Nf‐H (E), Nf‐L (F), Nf‐H Abs, and Nf‐L ICs (I) were added to the prognostic model. Vertical dashed line denotes unchanged mortality (HR = 1). Closed circle denotes median HR and red whiskers 95% CI. Pairwise Kaplan–Meier survival analysis from V1 of ALS patients from ABC (C) and PGB (D) with Nf‐H Abs plasma concentration above Q3 (higher levels and upper quartile) and below Q3. Kaplan–Meier survival analysis was performed in ABC (G) and in PGB (H) using Nf‐H protein plasma concentration above and below median. The effect of Nf‐L plasma levels above and below median was also tested in the ABC (J) and PGB (K) cohorts. Red lines indicate ALS subgroups with higher analyte level (above median or Q3) and black lines ALS subgroups with lower level (below median or Q3). *p* value was obtained from Log‐rank test chi square; *p* ≤ 0.05 was considered to be statistically significant. Log‐rank Chi‐square and *p* values as well as survival in months calculated for each subset of ALS patients are reported for each Kaplan–Meier figure.

Using univariate Kaplan–Meier and multivariate Cox proportional hazard analyses we investigated the ability of Nf Abs and ICs plasma levels to predict the survival in ALS subgroups compared to Nf‐L and Nf‐H proteins. ALS patients were dichotomized according to cut offs of Nf and Nf Abs/ICs plasma concentrations including the median (e.g., above and below median) and third quartile (Q3; >75 percentile for higher levels). Clinical, genetic, and demographic covariates previously associated with survival in ALS were incorporated in the Cox survival model and initially tested as independent predictors of survival, including bulbar onset of the disease, presence of a *C9orf72* mutation, ΔFRS, sex (female), and age at baseline (V1). Higher plasma levels of Nf‐L, Nf‐H, Nf‐Abs, and Nf‐ICs were then incorporated into the prognostic model. The time lines in the survival models were disease duration from V1 and from symptom onset (Fig. 4).

We observed a significantly reduced survival in ALS patients with Nf‐L and Nf‐H concentrations above the median and Q3 (above median *p* = 0.0011 for Nf‐H and *p* < 0.0001 for Nf‐L, Fig. 4G and J; above Q3, *p* = 0.0018 for Nf‐H and *p* < 0.0001 for Nf‐L Fig. 4H and K; survival time from V1). Nf and DPR Abs and ICs plasma concentrations above and below the median were not associated with survival (data not shown). There was a significant association between Nf‐H Abs above Q3 and survival in both the PGB and ABC (*p* = 0.0231 and *p* = 0.0065; Fig. 4C and D).

As previously reported,^9^ the Cox proportional hazard model for clinical variables only showed a strong association between progression rate at V1 (ΔFRS) and survival from both V1 and from disease onset (HR: 2.893, CI: 2.231–3.921 and HR: 13.748 CI: 8.195–23.026, respectively, *p* < 0.0001; Fig. 4A and B). Similarly, age at V1, ALSFRS‐R at V1, bulbar onset, and C9+ve status had associations with survival when calculated from disease onset (*p* = 0.046, *p* = 0.005, *p* = 0.039, and *p* = 0.001, respectively, Fig. 4A and B).

Adding Nf‐H and Nf‐L protein concentrations above the median to the multivariate prognostic model showed that higher levels of these analytes had a mild but significant effect on survival only for Nf‐H (HR: 1.728, CI: 1.026–2.909, *p* = 0.040), while Nf‐L protein concentration effect was only close to significance (HR: 1.921, CI: 0.986–4.056, *p* = 0.068; Fig. 4E and F). Inclusion of Nf‐H Abs and Nf‐L ICs (both above median and above Q3), individually or in combination, did not alter the prognostic model (Fig. 4G). Expression levels of Nf‐M Abs and poly‐(GP) DPR Abs were not good predictors of survival (data not shown).

### Correlations among analyte levels and clinical variables

Pairwise associations between plasma concentration of the target analytes at baseline and between the same analytes and functional measures of disease progression were tested using Pearson’s correlation analysis (Table S1).

No correlation was observed between total Nf levels and total Nf Abs or Nf ICs. A weak positive correlation was observed between Nf‐H proteins and Nf‐H Abs (*R* = 0.22, *p* = 0.013; Table S1). Nf‐L showed a moderate to strong correlation with parameters of clinical progression including ALSFRS‐R change (*R* = 0.466, *p* = 0.0004), ΔFRS (*R* = 0.502, *p* = 0.0001), disease duration (*R* = −0.491, *p* = 0.0002), and ALSFR‐S (*R* = −0.382, *p* = 0.0044, Table S1). In line with the observed association between higher Nf‐H Abs and shorter survival, the mean of the longitudinal Nf Abs levels was found to correlate with ΔFRS (*R* = 0.4174, *p* = 0.0339) and inversely with ALSFRS‐R (*R* = −0.4354, *p* = 0.0262). Baseline Nf Abs levels also correlated inversely with disease duration from onset *(R* = −0.358, *p* = 0.0012) in ALS patients (ABC). These findings were generally confirmed in the PGB cohort, where a positive association was found between Nf‐H Abs and Nf‐L ICs at baseline and ΔFRS (*R* = 0.2022, *p* = 0.0216 and *R* = 0.4590, *p* = 0.041, respectively; Table S1).

Only Nf‐H plasma concentration showed a positive correlation with age (*R* = 0.2654, *p* = 0.0371).

### Longitudinal analysis

Changes in concentration of the markers of humoral response to Nf in the longitudinal samples from ALS patients with a faster, slower disease progression, and with a genetic mutation of the *C9orf72* gene were investigated.

**Figure 5:**
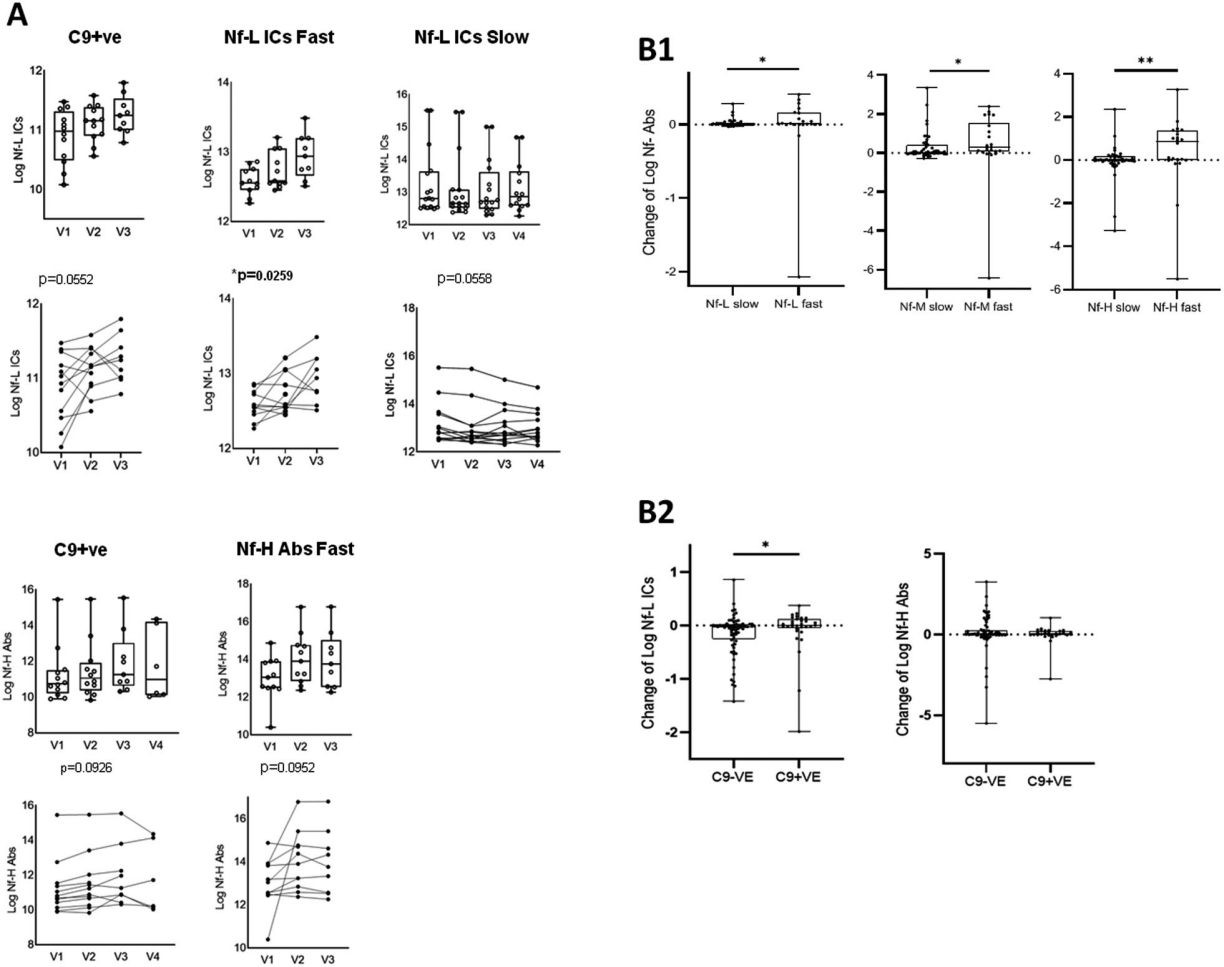
Changes of neurofilaments (Nf) Abs and ICs between time points. (A) Box plot (above) and spaghetti plot (below) representation of ANOVA for repeated measures (mixed model with missing values at random) analysis of the changes from V1 to last time point of Nf‐L ICs, Nf‐H Abs in ALS patients stratified according to rate of disease progression (ALS‐F and ALS‐S) and presence of C9orf72 genetic mutation (C9+ve). Only markers with a statistically significant change over time (or close to statistical significance) are represented in which the remaining ANOVA repeated measures analyses are reported in the Figure S2. (B1): Box plots showing the change of neurofilament Abs expression levels (to Nf‐L, Nf‐M, and Nf‐H isoforms) over time in ALS individuals with a faster (ALS‐F) and slower (ALS‐S) progression of the disease and (B2) of Nf‐H Abs and Nf‐L ICs in ALS individuals with a C9orf72 genetic mutation (C9+ve) compared to non‐mutation carriers (C9‐ve). Abs and ICs change is expressed as the log‐transformed difference between last time point and V1 in relation to the time interval between the time points. ALS‐F present higher levels of analyte change: Nf‐L Abs (*p* = 0.0357), Nf‐M Abs (*p* = 0.0193), and Nf‐H Abs (*p* = 0.0043). Box and whiskers plots show the 25th and 75th percentiles, median line, and outliers.

We found a significant upregulation of Nf‐L ICs in T2 and T3 compared to T1 (*p* = 0.0259) and the same trend of increase (close to statistical significance) for Nf‐L ICs and Nf‐H Abs in C9+ve ALS and in ALS‐F, respectively (Fig. 5A). In contrast, Nf‐L ICs appeared to decrease over time in ALS‐S (Fig. 5A).

Changes of concentration between Tlast and T1 for Nf‐L, Nf‐M, and Nf‐H Abs were higher in faster compared to slower progressing ALS individuals (Nf‐L Abs, *p* = 0.011; Nf‐M Abs *p* = 0.017 and Nf‐H Abs *p* = 0.01; Fig. 5B1) while Nf‐L ICs were higher in C9+ve compared to C9‐ve ALS patients (Fig. 5B2, *p* < 0.05).

## Discussion

We show that like heavy‐ and light‐chain Nf proteins, Nf‐H Abs and Nf‐L ICs have a significantly higher concentration in blood from ALS individuals compared to HC, which is particularly elevated in faster progressing individuals (Fig. 5). This study also shows that Nf‐L and Nf‐H proteins are the best‐performing biomarkers in the separation of ALS from HC and faster from slower progressing ALS, in line with previously reported data.^8^, ^25^, ^26^

The analysis of Nf proteins along with their Abs, the biomarkers under investigation in this study, provides further insight into the use of both antigen and Abs as disease biomarkers for ALS. Critically, we observe a trend of increasing concentrations of plasma Nf‐L ICs and Nf Abs in the longitudinal follow‐up of fast progressing ALS and in ALS patients with a *C9orf72* mutation (Fig. 5). This upward trend diverges from the reported relative stability of Nf proteins in ALS individuals in longitudinal measurements.^7^, ^10^ One possible explanation of the relatively unchanged Nf levels and of raising Abs in the progression of the disease, is the production of Nf Abs with a progressively higher antigen affinity, leading to a more effective clearance of these axonal proteins in a later disease stage, as neuro‐axonal destruction gathers momentum. This change of adaptive immunity would ultimately level out Nf concentration over time.

The study of the antibody response to core axonal proteins like Nf may also further our understanding of the ALS pathobiology. It is possible that the blood–brain barrier (BBB) endothelial cell damage described in ALS26^27^, ^28^ facilitates access of Abs and ICs against Nf and DPR into the CNS where they may have a detrimental effect on degenerating neurons. While this interpretation remains highly speculative, it is important to observe that a toxic effect of Nf‐L Abs has already been shown in vitro using neuronal cell cultures, along with a significant worsening of the pathological phenotype of experimental encephalomyelitis following treatment of animal models with the same Abs.^29^, ^30^

It is also possible to hypothesize that the formation of Abs to Nf‐H proteins in the peripheral circulation may function as a sink and speed up Nf‐H proteins elimination. This humoral response would ultimately shift the equilibrium dynamics of Nf‐H proteins across a more permeable BBB to the periphery and away from the areas of pathological protein aggregation. Our recent observation that Nf‐H proteins are sequestered into circulating aggregates in blood may point toward this way of disposing of Nf‐H.^25^, ^31^ This concept has been recently proposed for the A‐Beta pathological burden in animal models of Alzheimer’s disease.^32^ Similarly, levels of naturally occurring Abs with high affinity to α‐synuclein have been shown to be reduced in Parkinson’s disease patients compared to HC, suggesting a failure of this protective sink for toxic peptides under pathological conditions.^33^

We also report and elevated immune response to poly‐(GP) DPR in C9+ve compared to C9‐ve ALS patients. The expression of poly‐(GP) peptides in CSF from C9+ve ALS patients and cell lines has recently emerged as a potential pharmacodynamic and target engagement biomarker in C9+ve ALS patients.^16^ Poly‐(GP) peptide levels remain relatively stable from the pre‐symptomatic to a clinically manifest stage of ALS and are only measurable in CSF.^16^ In contrast, in our study, both Nf‐H Abs and Nf‐L ICs in blood appear to increase longitudinally in all symptomatic C9+ve ALS patients, individuals with an intermediate rate of disease progression compared to faster and slower ALS patients. Crucially for the development on novel ALS therapeutics, our data suggest that the detection in blood of epitope‐specific Nf and poly‐(GP) DPR Abs/ICs could provide more easily accessible biomarkers for the selection of faster progressing and C9+ve ALS cases in clinical trials targeting these sub‐sets of ALS patients.^15^

This study confirms the relevance of Nf‐L and Nf‐H proteins as blood biomarkers for the clinical stratification of ALS. However, we also show the potential for a biomarker built on the humoral response to Nf, which is abundant and cost‐effectively measurable in accessible biological fluids. Samples for Abs/ICs detection could in fact be collected remotely using newly developed methods of remote microsampling, including dry plasma spots^34^ and developed alongside sub‐picomolar sensitivity methods for the detection of neurofilament proteins. Future experiments will have to be conducted using larger longitudinal cohorts, including pre‐symptomatic ALS individuals, but also focus on immunoglobulins isotypes as well as on their target affinity in relation to disease progression. Additionally, it will be important to calibrate future experiments on the detection of more specific immune responses, using proteins that reproduce the whole range of posttranslational modifications that have been described in ALS.

## Author Contributions

FP and AM contributed to the conception and design of the study. All authors contributed to the design of the study; FP and VL contributed to the acquisition and analysis of data; YB contributed to sample processing and data collection; CH, FP, and AM performed the statistical analysis; OY and AM contributed with the recruitment of patients and controls and data collection process; PF and AI performed the genetic analysis; JW contributed to data management and CReATe biorepository sample selection and matching and preparation of clinical data for analysis; MB contributed to the recruitment and evaluation of patients; FP, VL, CL, OY, PF, AI, YB, JW, MB, and AM contributed to data interpretation and discussion, writing, and revision of the manuscript.

## Conflict of Interest

Nothing to report.

## Funding Information

The Post‐Doctoral Research Assistant involved in the experimental has been supported for 1 year by EU2020 funding (H2020 PHC‐13‐2014): “Efficacy and safety of low‐dose IL‐2 (ld‐IL‐2) as a Treg enhancer for anti‐neuroinflammatory therapy in newly diagnosed Amyotrophic Lateral Sclerosis (ALS) patients (MIROCALS)”. Research staff (Ozlem Yildiz) working for the research project: “A Multicentre Biomarker Resource Strategy in ALS (AMBRoSIA)” has also provided support to the study and helped in the design and conduct of experiments. The authors also thank the CReATe Consortium (U54NS092091) for the support and access to biological samples and clinical information. The CReATe Consortium is part of the Rare Diseases Clinical Research Network (RDCRN), an initiative of the Office of Rare Diseases Research (ORDR), NCATS. CReATe is funded through a collaboration between NCATS and the NINDS.

## Supporting information


**Figure S1**. (A): Group analysis comparing the levels of Nf‐M Abs in healthy individuals (HC) and phenotypic (ALS‐F and ALS‐S) variants of ALS. Higher Nf‐M Abs were observed in ALS (*p* = 0.033), particularly in ALS‐F (*p* = 0.04) compared to HC. No significant differences in Nf‐L Abs levels were observed between variants of ALS. Kruskal–Wallis one‐way analysis was used for multiple comparisons. The scatter dot plots show the median with interquartile range. The statistical difference between groups is shown as (*p* ≤ 0.05). (B): Receiver operating characteristic (ROC) nonparametric analysis was used to assess the ability of the analytes to discriminate ALS phenotypic subgroups from HC (plots indicate sensitivity against 1‐specificity). ALS‐F from HC (AUC = 0.64, *p* = 0.011) and ALS from HC (AUC = 0.63, *p* = 0.0056).

**Figure S2**. Changes of neurofilaments (Nf) Abs and ICs between time points. Box plot (above) and spaghetti plot (below) representation of ANOVA for repeated measures (mixed model with missing values at random) analysis of the changes from V1 to last time point of Neurofilament Abs and immune complexes in ALS patients stratified according to rate of disease progression (ALS‐Fast and ALS‐Slow).

**Table S1**. Correlation analysis in the cross‐sectional cohort studies: ABC, ALS biomarkers study cohort and PGB, phenotype–genotype biomarker study. Table shows the Pearson’s analysis correlation between levels of neurofilaments antibodies (Abs), immune complexes (ICs), Nf protein and poly‐(GP) antibodies and age, scores of disease progression and functional impairment ALS C9+ve and C9‐ve patients. Pearson’s correlation between baseline neurofilaments antibodies (Abs) and immune complexes (ICs) and scores of disease progression and functional impairment. Nf, neurofilaments; Poly‐(GP) DPR, poly‐GP dipeptide repeats; ALSFRS‐R, Amyotrophic lateral sclerosis functional rating scale‐revised score at baseline; ΔFRS, delta FRS progression rate using the total ALSFRS‐R and disease duration; ALSFRS‐R change, magnitude of change in ALSFRS over time; Abs, antibodies; ICs, immune complexes; ALS C9+ve, C9orf72 mutation carriers; ABC, ALS biomarkers study cohort; PGB, phenotype–genotype biomarker study.
