# Supplementary figures and images for: Humoral response to neurofilaments and dipeptide repeats in ALS progression
Source: Ann Clin Transl Neurol. 2021 Jul 27;8(9):1831–44. doi: 10.1002/acn3.51428 (PMC8419401; doi:10.1002/acn3.51428)

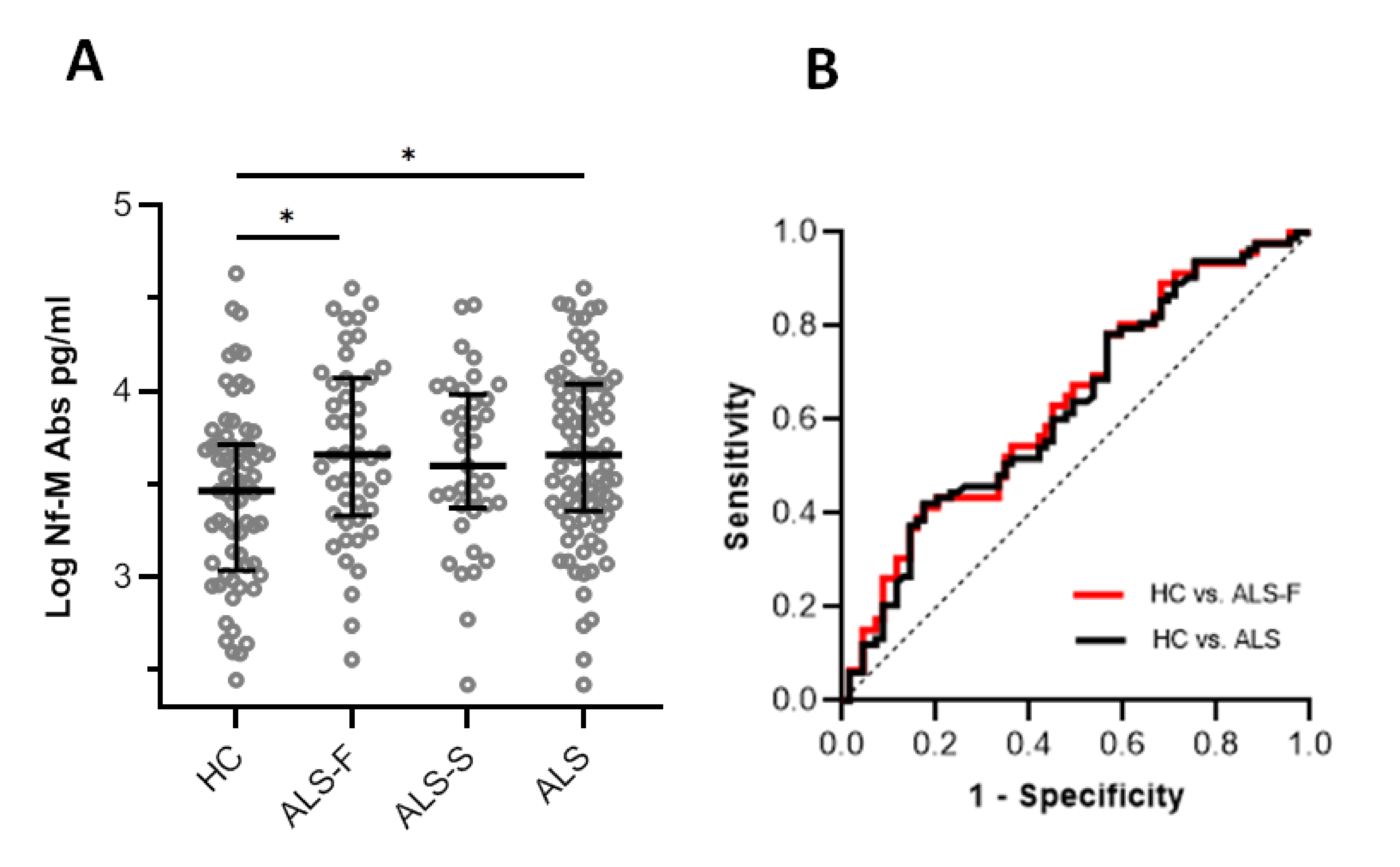

Supplement: Supplementary file 1 — Figure S1. (A): Group analysis comparing the levels of Nf‐M Abs in healthy individuals (HC) and phenotypic (ALS‐F and ALS‐S) variants of ALS. Higher Nf‐M Abs were observed in ALS (p = 0.033), particularly in ALS‐F (p = 0.04) compared to HC. No significant differences in Nf‐L Abs levels were observed between variants of ALS. Kruskal–Wallis one‐way analysis was used for multiple comparisons. The scatter dot plots show the median with interquartile range. The statistical difference between groups is shown as (p ≤ 0.05). (B): Receiver operating characteristic (ROC) nonparametric analysis was used to assess the ability of the analytes to discriminate ALS phenotypic subgroups from HC (plots indicate sensitivity against 1‐specificity). ALS‐F from HC (AUC = 0.64, p = 0.011) and ALS from HC (AUC = 0.63, p = 0.0056). [file ACN3-8-1831-s001.tif]
