# Supplementary material for: Humoral response to neurofilaments and dipeptide repeats in ALS progression
Source: Ann Clin Transl Neurol. 2021 Jul 27;8(9):1831–44. doi: 10.1002/acn3.51428 (PMC8419401; doi:10.1002/acn3.51428)
Supplement: Supplementary file 3 — Table S1. Correlation analysis in the cross‐sectional cohort studies: ABC, ALS biomarkers study cohort and PGB, phenotype–genotype biomarker study. Table shows the Pearson’s analysis correlation between levels of neurofilaments antibodies (Abs), immune complexes (ICs), Nf protein and poly‐(GP) antibodies and age, scores of disease progression and functional impairment ALS C9+ve and C9‐ve patients. Pearson’s correlation between baseline neurofilaments antibodies (Abs) and immune complexes (ICs) and scores of disease progression and functional impairment. Nf, neurofilaments; Poly‐(GP) DPR, poly‐GP dipeptide repeats; ALSFRS‐R, Amyotrophic lateral sclerosis functional rating scale‐revised score at baseline; ΔFRS, delta FRS progression rate using the total ALSFRS‐R and disease duration; ALSFRS‐R change, magnitude of change in ALSFRS over time; Abs, antibodies; ICs, immune complexes; ALS C9+ve, C9orf72 mutation carriers; ABC, ALS biomarkers study cohort; PGB, phenotype–genotype biomarker study. [file ACN3-8-1831-s003.docx]

**Supplementary Table 1**

| **Analytes**  **(cross-sectional cohorts)** | **Age at baseline** | **Nf-L** | **Nf-M** | **Nf-H** | **Total Nf proteins** | **ALSFRS-R**  **change** | **Δ FRS** | **Disease duration from onset** | **ALSFRS-R** |
| --- | --- | --- | --- | --- | --- | --- | --- | --- | --- |
| **Total Nf Abs** | R= 0.210  P=0.059 | n/a | n/a | n/a | R= 0.148  P=0.071 | R= **-**0.0161  P=0.886 | R= 0.0121  P=0.915 | R= **-**0.199  P=0.0762 | R= **-**0.0102  P=0.363 |
| **Nf-L Abs** | R= 0.133  P=0.244 | R= 0.178  P=0.208 | n/a | n/a | n/a | R= **-**0.0552  P=0.629 | R= 0.0747  P=0.513 | R= **-**0.0264  P=0.819 | R= **-**0.148  P=0.192 |
| **Nf-M Abs** | R= 0.162  P=0.151 | n/a | R= 0.120  P=0.314 | n/a | n/a | R= **-**0.0492  P=0.665 | R= **-**0.122  P=0.281 | R= **-**0.175  P=0.123 | R= 0.0122  P=0.915 |
| **Nf-H Abs** | R= 0.047  P=0.674 | n/a | n/a | R= 0.220  P=0.0132 | n/a | R= 0.182  P=0.106 | R= 0.116  P=0.304 | R= **-**0.263  P=0.019 | R= 0.0147  P=0.915 |
| **Nf-H Abs (ABC)** | R=0.0839  P=0.4593 | n/a | n/a | n/a | n/a | R= **-**0.2571  P=0.0213 | R= 0.2051  P=0.062 | R= **-**0.3586  P=0.0012 | R= **-**0.2122  P=0.902 |
| **Nf-H Abs (PGB)** | R= 0.127  P=0.174 | n/a | n/a | n/a | n/a | R= **-**0.0084  P=0.924 | R= 0.2022  P=0.0216 | R= 0.045  P=0.9867 | R= 0.077  P=0.3855 |
| **Total Nf ICs** | R= **-**0.132  P=0.241 | n/a | n/a | n/a | R= **-**0.120  P=0.296 | R= 0.163  P=0.147 | R= 0.0817  P=0.468 | R= **-**0.0026  P=0.981 | R= **-**0.0509  P=0.652 |
| **Nf-L ICs** | R= **-**0.132  P=0.241 | R= **-**0.090  P=0.522 | n/a | n/a | n/a | R= 0.155  P=0.170 | R= 0.18  P=0.341 | R= 0.0074  P=0.499 | R= **-**0.0767  P=0.499 |
| **Nf-M ICs** | R= **-**0.0787  P=0.485 | n/a | R= 0.029  P=0.804 | n/a | n/a | R= **-**0.155  P=0.166 | R= **-**0.146  P=0.193 | R= **-**0.108  P=0.342 | R= 0.118  P=0.296 |
| **Nf-H ICs** | R = **-**0.0215  P=0.849 | n/a | n/a | R = **-**0.095  P=0.462 | n/a | R= 0.118  P=0.293 | R= **-**0.0410  P=0.716 | R= **-**0.0307  P=0.787 | R= 0.0935  P=0.406 |
| **Nf-L ICs (PGB)**  ALS C9-ve | R = **-**0.0345  P=0.721 | n/a | n/a | n/a | n/a | R= **-**0.0933  P=0.334 | R= 0.0815  P=0.394 | R= 0.0835  P=0.387 | R= 0.047  P=0.596 |
| **Nf-L ICs (PGB)**  ALS C9+ve | R = **-**0.0616  P=0.796 | n/a | n/a | n/a | n/a | R= 0.1865  P=0.431 | R= 0.4590  P=0.0418 | R= **-**0.1459  P=0.539 | R= **-**0.0042  P=0.986 |
| **Poly (GP) DPR Abs (ABC)**  ALS C9-ve |  | n/a | n/a | n/a | n/a | R= **-**0.0933  P=0.4193 | R= **-**0.0853  P=0.4606 | R= 0.0285  P=0.80 | R= **-**0.0090  P=0.937 |
| **Poly (GP) DPR Abs (ABC)**ALS C9+ve |  | n/a | n/a | n/a | n/a | n/a | R= 0.0069  P=0.974 | R= **-**0.2453  P=0.237 | R= 0.1430  P=0.505 |
| **Poly (GP) DPR Abs (PGB)**ALS C9-ve | R = **-**0.197  P=0.102 | n/a | n/a | n/a | n/a | R= 0.1580  P=0.191 | R= 0.0856  P=0.654 | R= **-**0.0190  P=0.875 | R= **-**0.0340  P=0.780 |
| **Poly (GP) DPR Abs (PGB)**  ALS C9+ve | R= 0.0906  P=0.712 | n/a | n/a | n/a | n/a | R= 0.0386  P=0.875 | R= **-**0.410  P=0.0813 | R= 0.2211  P=0.362 | R= 0.0894  P=0.716 |
| **Nf-L protein** | R=0.2171  P=0.1149 | n/a | n/a | n/a | n/a | R= 0.466  P=0.0004 | R= 0.502  P=0.0001 | R= **-**0.491  P=0.0002 | R= **-**0.382  P=0.0044 |
| **Nf-H protein** | R=0.2654  P=0.0371 | n/a | n/a | n/a | n/a | R= 0.376  P=0.0026 | R= 0.179  P=0.164 | R= **-**0.288  P=0.0246 | R= **-**0.312  P=0.0135 |

Correlation analysis (Pearson’s) between baseline neurofilaments antibodies (Abs) and immune-complexes (ICs) and scores of disease progression and functional impairment.

**Nf:** neurofilaments; **Poly-(GP) DPR:** poly-GP dipeptide repeats; **ALSFRS-R**: Amyotrophic lateral sclerosis Functional Rating Scale Revised score at baseline; **ΔFRS**: Delta FRS progression rate using the total ALSFRS-R and disease duration; **ALSFRS-R change**: magnitude of change in ALSFRS over time; **Abs**: Antibodies; **ICs**: Immune complexes; **ALS C9+ve :** C9orf72 mutation carriers**; ABC**: ALS biomarkers study cohort; **PGB**: Phenotype Genotype Biomarker study.
